# Rely on Each Other: DNA Binding Cooperativity Shapes p53 Functions in Tumor Suppression and Cancer Therapy

**DOI:** 10.3390/cancers13102422

**Published:** 2021-05-17

**Authors:** Oleg Timofeev, Thorsten Stiewe

**Affiliations:** Institute of Molecular Oncology, Universities of Giessen and Marburg Lung Center (UGMLC), Member of the German Center for Lung Research (DZL), Philipps-University, 35037 Marburg, Germany

**Keywords:** DNA binding cooperativity, p53, mutation

## Abstract

**Simple Summary:**

p53 is a DNA-binding protein that activates hundreds of genes, which act concertedly to suppress the development, expansion, and spreading of cancer cells. The remarkable tumor suppressive activity relies on p53′s ability to bind DNA not as a single molecule, but in a cooperative manner as a complex of four tightly interacting proteins. We describe the structural features of p53 that enable DNA binding cooperativity and review the implications for p53 function. In approximately 50% of cancers, p53 is inactivated by mutations that either distort the 3D structure of the protein or destroy points of DNA contact. In this review, we emphasize that an estimated number of 34,000 cancer cases annually are caused by a third class of so-called “cooperativity” mutations, which selectively compromise the cooperative nature of DNA binding. We highlight the unique characteristics of tumors with p53 cooperativity mutations and discuss personalized treatment options for these cancer patients.

**Abstract:**

p53 is a tumor suppressor that is mutated in half of all cancers. The high clinical relevance has made p53 a model transcription factor for delineating general mechanisms of transcriptional regulation. p53 forms tetramers that bind DNA in a highly cooperative manner. The DNA binding cooperativity of p53 has been studied by structural and molecular biologists as well as clinical oncologists. These experiments have revealed the structural basis for cooperative DNA binding and its impact on sequence specificity and target gene spectrum. Cooperativity was found to be critical for the control of p53-mediated cell fate decisions and tumor suppression. Importantly, an estimated number of 34,000 cancer patients per year world-wide have mutations of the amino acids mediating cooperativity, and knock-in mouse models have confirmed such mutations to be tumorigenic. While p53 cancer mutations are classically subdivided into “contact” and “structural” mutations, “cooperativity” mutations form a mechanistically distinct third class that affect the quaternary structure but leave DNA contacting residues and the three-dimensional folding of the DNA-binding domain intact. In this review we discuss the concept of DNA binding cooperativity and highlight the unique nature of cooperativity mutations and their clinical implications for cancer therapy.

## 1. Introduction

**Figure 1:**
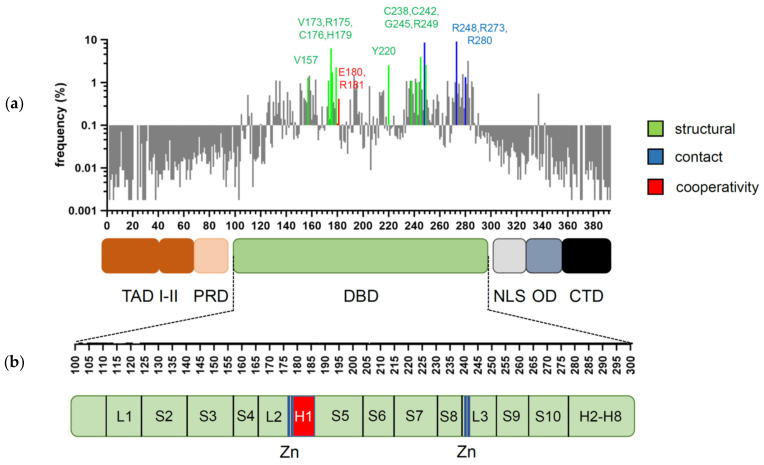
Domain structure of p53 and distribution of cancer-associated mutations. (**a**) Frequency and distribution of *TP53* mutations detected in somatic tumors. Highlighted are the codons most frequently affected by structural (green), contact (blue), and cooperativity (red) mutations (references in text). (**b**) Secondary structure of p53 DBD. Residues responsible for zinc (Zn) ion coordination (blue) and H1 helix (red) are highlighted.

p53 is a transcription factor that has its evolutionary origin in unicellular choanoflagellates and early metazoans [1]. Its functions in protection of genome integrity in somatic cells have crystallized later in vertebrate organisms, where it evolved into a tumor suppressor and deserved recognition as one of the most powerful ones [2]. The human *TP53* gene can give rise to several splice variants [3]. Among them, the full-length isoform p53α is the most abundant and best characterized. It consists of 393 amino acids and can be subdivided into several functional domains (Figure 1a,b): two N-terminal transactivation domains (TADI and II, amino acids 1–43 and 43–63); a short proline-rich domain (PRD, aa 64–93), involved in protein–protein interactions and apoptosis; a core DNA-binding domain (DBD, aa 94–292) that enables sequence-specific DNA binding; a hinge region (aa 293–323) where a bipartite nuclear localization signal (NLS) is located; an oligomerization domain (OD aa 324–355) that mediates tetramerization of p53 molecules; and a carboxy-terminal regulatory domain (CTD aa 356–393), which binds DNA in a sequence non-specific manner and regulates p53 function and stability [1,4,5].

p53 undergoes complex post-translational modifications that determine its stability, cellular localization, and transcriptional activity [6]. Upon stress, specific phosphorylation and acetylation patterns of the TAD and CTD are set and lead to stabilization and activation of p53 [7], which cooperatively acts as a homotetramer in sequence-specific DNA binding and transcriptional regulation. Numerous target genes trigger a range of cellular programs—from repair of cellular damage to elimination of harmed cells, depending on stress impact and damage level [8]. Besides transcriptional functions, p53 exerts transcription-independent activities in regulation of apoptosis [9], necrosis [10], autophagy [11], metabolism [12,13], and DNA replication [14] and repair [15]. Multiple p53-controlled effector mechanisms add to its power as a tumor suppressor and create strong selective pressure against p53 in tumorigenesis.

Mutations, that hit the *TP53* gene are found in virtually all cancer types, but the frequency of inactivating mutations may vary from less than 5% in neuroblastoma to above 90% in ovarian and small-cell lung cancer [16,17,18,19,20]. Among all genetic alterations that affect *TP53* in tumors, missense mutations prevail, the majority of which cluster in the central DBD, underscoring its significance for p53′s tumor suppressive activity [16,20] (Figure 1). Mutations that occur with an overall frequency >1% are considered as “hotspots” and together represent about 30% of all missense mutations [21,22]. The role of these hotspot mutations for p53 structure and function is well described [23,24]. Several high-throughput screens were undertaken to investigate the remaining 70% of p53 variants [25,26,27], but the precise mechanisms how they affect p53 are less well understood. With respect to transcriptional and tumor-suppressive p53 activities, the majority are loss-of-function (LOF) mutations, which, however, affect p53 to different degrees—from partial impairment to complete inactivation. Furthermore, several hotspot mutations have been shown to bestow p53 with neomorphic, oncogenic functions and are commonly referred to as gain-of-function (GOF) mutants, which promote tumor progression, metastasis, and resistance to therapy.

Since a number of recent reviews provide comprehensive information about these aspects of p53 mutations [22,28,29], we focus this review on a new class of p53 mutations that undermine the cooperative nature of DNA binding and discuss the role of these mutations in cancer development and therapy response and implications for p53 functions in tumor suppression.

## 2. Cooperative DNA Binding by p53

**Figure 2:**
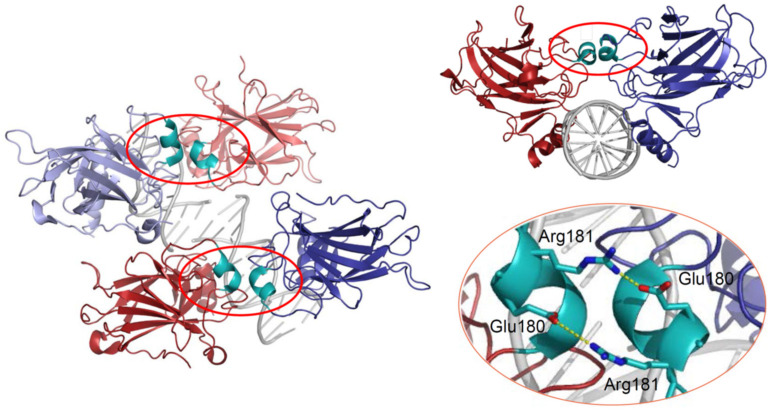
3D structure of the p53 DBD tetramer in complex with DNA (based on RCSB Protein Data Bank ID:2AHI). H1 helices are highlighted in cyan; ionic bonds between negatively charged Glu180 and positively charged Arg181 are shown in yellow.

p53 can bind DNA in two modes: sequence-specifically via the DBD and in a sequence-independent manner through the CTD [30]. Like many other transcription factors, p53 forms oligomers, and the transcriptionally active state of p53 requires the assembly of homotetramers [31]. In vitro experiments suggested that p53 dimers are assembled co-translationally and are predominant under basal conditions [32,33,34], whereas stress-induced post-translational modifications promote tetramerization [35,36]. A recent in vivo study has shown that in non-stressed cells monomers and dimers composed the major p53 pool, but DNA damage triggered rapid tetramerization [37]. The ability of p53 to bind double-stranded DNA in a sequence-specific manner is determined by the DBD, which forms an immunoglobulin-like β-sandwich structure composed of a loop-sheet-helix motif and two large loops (L2 and L3) and serves as a basic scaffold for the DNA-binding surface. The loop-sheet-helix motif that includes the L1 loop binds the minor groove, whereas the L2 and L3 loops, stabilized by a zinc ion, dock to the major groove [31,38]. A typical p53 response element (RE) contains two decameric RRRCWWGYYY (R = A,G; W = A,T; Y = C,T) half-sites separated by spacers of 0–18 base pairs (bp) [39,40]. Structural studies demonstrated that wild-type p53 proteins form tetrameric complexes on DNA, known as “dimers of dimers”, where each clamp-like symmetrical dimer binds one half-site of the RE, and two dimers occupy the full RE (Figure 2) [31,41,42,43,44,45,46].

Assembly of p53 tetramers on DNA occurs in a highly cooperative manner. In general, cooperativity is a common biochemical phenomenon observed when a protein or protein complex contains multiple identical or near-identical ligand binding sites, and ligand binding to any one site increases (positive cooperativity) or decreases (negative cooperativity) the apparent affinity at the others. Cooperative binding can be mathematically described by the Hill equation, where the Hill coefficient functions as a quantitative measure of cooperativity [47]. One of the best-known examples of positive cooperativity is oxygen binding by hemoglobin, where binding of each oxygen molecule increases the binding affinity for the next until the hemoglobin tetramer is fully saturated with oxygen at all four binding sites [48]. Many other molecular assemblies exhibiting cooperative binding have been studied, including multimeric enzymes and transcription factors such as lambda phage repressor. In a very similar manner, DNA binding of p53 also displays positive cooperativity relying on the assembly of a tetrameric p53–DNA complex that is stabilized by protein–protein interactions between the four monomers [41]. Primarily, tetramerization of p53 monomers is mediated by the OD, which consists of a short β-strand and an α-helix that provide an interaction surface for dimerization. Two primary dimers associate through their α-helices and build a four-helix bundle, stabilized by hydrophobic interactions [49]. Integrity of the OD is essential for tetramerization and p53 functional activity. However, it has been shown that isolated DBDs upon interaction with p53 REs form tetramers also independent of the OD, although with 10- to 1000-fold lower binding affinity [34,50,51]. These findings indicated existence of direct protein–protein interactions between DBDs. These interactions among p53 dimers and tetramers stabilize protein/DNA complexes and shape p53′s cistrome and transcriptional activity [52,53]. Early structural models of p53 DBD/DNA complexes pointed at the H1 helix as the structural basis for cooperative interactions between core domains [31,51]. Later models based on X-ray crystallography and NMR data indicated that the DBD interaction interface is formed by residues from the H1 helix (Pro177, His178, Glu180, Arg181) and several residues from the L3 loop (Met243, Gly244) [44,54]. The H1 helix is a short α-helical structure (Pro177-Cys182) located within the L2 loop, adjacent to the DNA-binding core region but not involved in direct contact with DNA (Figure 2). Association of p53 with its RE brings the antiparallel oriented H1 helices of monomers in proximity and allows interactions that stabilize the entire complex. X-ray crystallography, NMR, and biochemical and biophysical studies pointed to an essential role of these structures for cooperative DNA binding and identified Glu180 and Arg181 as key residues that mediate reciprocal electrostatic interactions between H1 helices [42,44,54,55,56,57]. The double salt bridges formed by these oppositely charged amino acids maintain p53′s intra-dimer interactions and affect the strength of sequence-specific DNA binding [52,55,56,58]. Intriguingly, the CTD that binds DNA in a sequence-independent manner contributes to cooperativity by inducing conformational changes within the DBD that enhance sequence-specific binding [59]. The primary structure of the H1 helix is highly conserved among p53 proteins of different vertebrate species and in the p53 family members p63 and p73, but the salt bridge itself is absent in p63 and p73 [55,57,60,61].

The degree of DNA binding cooperativity is determined by structural DNA properties encoded in the RE sequence—especially in its central WW dinucleotide [62]. Even though these bases are not directly contacted by p53 residues, they are highly conserved and determine the torsional flexibility of the half-site and thus define the energy needed for DNA twisting [62]. Since DNA binding of p53 induces significant RE bending and twisting, the exact WW dinucleotide sequence strongly affects binding [50,51,57,63]. In the case of a torsionally more flexible CATG, the p53 DBD binds with low cooperativity, while REs containing more rigid CAAG, and CTAG are bound with up to 3 orders of magnitude higher cooperativity and only when present in two contiguous p53 half-sites [62]. Additionally, REs containing spacers between two half-sites are bound with high cooperativity [62]. As transactivation relies on DNA binding and DNA-protein complex stability, cooperativity is a major determinant of p53′s transactivation function.

## 3. Cancer-Associated Mutations of the DBD—Structural Implications

The vast majority of cancer-associated mutations in the *TP53* gene are non-synonymous missense substitutions that give rise to more than 2000 mutant p53 variants [20,64]. Remarkably, over 95% of these mutations map to the core DBD [22]. The most frequent somatic “hotspot” mutations are R175H, R248Q/W, R273C/H, R282W, Y220C, G245S R249S, and V157F [20]. Based on the mechanism of action, mutations can be divided into three groups: structural, contact, and cooperativity mutations (Figure 1a).

### 3.1. Stuctural Mutations

In contrast to the intrinsically unfolded N- and C-terminal domains, the DBD is well structured. However, its stability is rather low: wild-type p53 denatures at 42–45 °C, and even at normal body temperature, the DBD in the context of the full-length protein has an unfolding half-life of 37 min, whereas the isolated DBD unfolds in just 9 min [65,66]. This low native stability of p53 makes it very sensitive to destabilizing mutations, which cause local or global unfolding. Many structural mutations that affect the β-sandwich region (such as V143A, V157F, Y220C, and F270C/L) or the loop-sheet-helix motif (R282W) are highly destabilizing: they reduce the thermodynamic stability of the protein by >3 kcal/mol, lowering the melting temperature by 5–7 °C [66,67,68]. As a result, the mutant protein is globally unfolded and unable to bind DNA at 37 °C but retains a wild-type-like conformation and substantial transcriptional activity at sub-physiological temperatures—so called “temperature-sensitive” mutants [69]. Because of the crucial role of the zinc ion for the core structure of the DNA-binding interface encoded by the L2-L3 loops, mutations that impact zinc binding also have a severely destabilizing effect. Non-hotspot mutations such as C176F, H179R, C238Y, and C242S directly affect zinc ligation, leading to a strongly reduced thermostability of the DBD [24,67]. The most frequent cancer mutation R175H heavily reduces zinc-binding affinity by destroying the zinc coordination sphere, which results in global unfolding at physiological and sub-physiological temperatures, making p53 completely inactive [70,71]. Other cancer-associated mutations, which hit this site with much lower frequency (such as R175C/L/P/S), seem to be less detrimental for zinc binding and have only moderate or weak effects on p53 functionality [24,72]. Mutations that affect the DNA-interacting surface can also cause local structural distortions that affect the DNA-binding proficiency of p53 to different degrees. The G245S and R249S hotspot mutations strike the L3 loop in the minor-groove-binding region. Whereas the G245S leads to small conformational changes (which, however, result in a substantial decrease in sequence-specific DNA binding), R249S has a more general impact on L3 loop conformation and DBD stability (it destabilizes the core domain by ∼2 kcal/mol), drastically affecting DNA binding [66,67,68].

### 3.2. Contact Mutations

Contact mutations most frequently affect the Arg248, Arg273, or Arg280 residues that are directly interacting with DNA. Arg248 is essential for docking into the minor groove and interacts with the regions flanking the core sequence of each half-site of RE [58]. Arg273 binds to the central CWWG site, providing important contacts to the DNA backbone. Arg280 anchors in the major groove, interacting with the conserved G in the CWWG sequence [42]. Arg248 and Arg273 are mutational hot-spots: R248Q/W and R273C/H substitutions represent 10–20% of all cancer-associated missense mutations detected in the DBD [20,22]. Contact mutations have only a minute effect on thermodynamic properties of the p53 protein and do not cause substantial structural perturbations [31,66,67] but drastically weaken sequence-specific DNA binding and thus disable p53′s transcriptional activity. For example, in vitro experiments with p53 tetramers containing DBD and OD showed that the R273H mutation reduced binding to the high affinity *GADD45* promoter by 1000-fold [66].

### 3.3. Cooperativity Mutations

**Figure 3:**
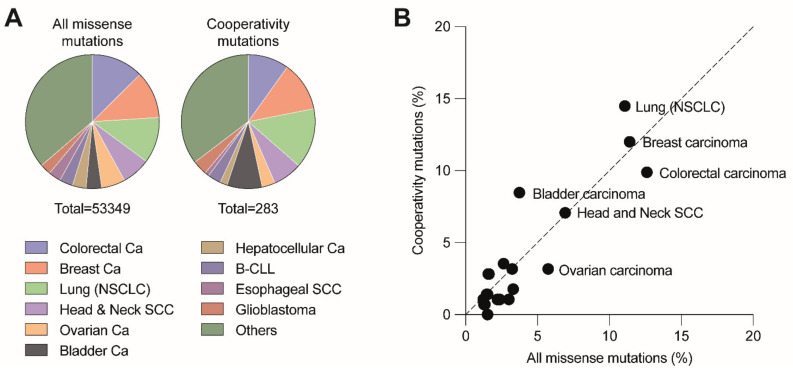
Distribution of cooperativity mutations across different cancer types. (**A**) Pie charts depict the distribution of all missense and cooperativity mutations across the listed cancer types. (**B**) Cancer type distribution of cooperativity mutations correlates with the distribution of all missense mutations (*R*^2^ = 0.8779, *p* < 0.001). Analysis based on all tumor samples with p53 missense mutations listed in the UMD TP53 Mutation Database (https://p53.fr/tp53-database, Release 2017_R2, accessed on 12 May 2021).

DBD mutations that affect the cooperative nature of p53 DNA binding are so-called “cooperativity” mutations. A number of cooperativity mutations at residues Glu180 (E180A/D/G/K/Q/V) and Arg181 (C/H/G/L/P/S) are found as somatic mutations in various types of sporadic cancer and germ-line mutations associated with the hereditary Li-Fraumeni or Li-Fraumeni-like cancer susceptibility syndromes. Together, these mutations account for 0.5–0.6% of all p53 missense mutations. Given that p53 is mutated in approximately 50% of all cancers and that 70% of these are missense mutations, this results in an estimated world-wide number of 34,000 cancer cases per year [20,22]. The distribution of cooperativity mutations across different cancer types is highly similar to all other missense mutations, showing only a slight overrepresentation of cooperativity mutations in non-small cell lung and bladder carcinoma and underrepresentation in colorectal and ovarian carcinoma (Figure 3). The most frequent cooperativity mutants (E180K, R181C, R181H) showed a selective loss of apoptosis, in parallel with reduced promoter binding and transactivation of apoptosis-related gene targets but retained substantial activity in mounting cell cycle arrest [52]. Another cooperativity mutant R181L, which is detected in somatic tumors and LFS patients, induced cell cycle arrest but failed to trigger apoptosis when ectopically expressed in p53-null cells [52,73].

In addition to these naturally occurring mutations, charge-neutralizing (E180L and R181L) and, in particular, charge-inverting (E180R and R181E) mutations were employed to experimentally weaken or disrupt the H1 helix salt bridges and delineate the functional impact of altered cooperativity. Of note, engineering charge-inversion mutations requires substitution of two (E180R) or three nucleotides (R181E), respectively. However, more than 99% of p53 missense mutations in cancer patients are single-nucleotide substitutions, providing an explanation for why these charge-inversion mutations have not been observed in cancer cells so far. Overlaying the NMR solution structure of charge-inverting mutations with the wild-type DBD showed differences in chemical shifts only for signals of residues within the H1 helix or near the specifically mutated residues [55]. Residues further away were only slightly affected by these mutations or not affected at all, which affords the conclusion that salt bridge mutants are folded in the native conformation and clearly distinguishes these cooperativity mutations from the other two classes of DBD mutations. Importantly, the DNA binding deficiency of these charge-inversion mutants is entirely rescued when the salt bridges are reconstituted by combination of the two mutant proteins (E180R+R181E) or introduction of both mutations into the same p53 molecule (E180R;R181E double mutant) [52,53,55,74]. This highlights that the cellular and organismal phenotypes resulting from these mutations are solely explained by the disruption of the H1 helix salt bridges and underlines the value of engineered charge-inversion mutations for studying the functional role of DNA binding cooperation in tumor suppression and beyond.

## 4. Functional Consequences of Cooperativity Mutations

### 4.1. Effects of Cooperativity Mutations on p53 DNA Binding

Initial experiments with the isolated p53 DBD demonstrated that the charge-inverting salt bridge mutations E180R and R181E lead to complete loss of sequence-specific DNA binding, similar to the hotspot contact mutation R248W [55]. More detailed analysis of codons 180 and 181 mutations in the context of full-length p53 tetramers revealed differences in DNA binding and p53/DNA complex stability for different mutants [52]. Whereas the charge-neutralizing mutation E180L only mildly reduced binding to the consensus site, the E180R had a significantly stronger effect but retained residual specific DNA binding activity. Consequences of R181 mutagenesis were even more pronounced, and the R181E mutant was completely unable to bind DNA. Importantly, and in line with the previous report [55], the combination of the complementary E180R and R181E mutants fully recovered binding to consensus sites, demonstrating the essential role of interactions between H1 helices for DNA binding and complex stabilization [52]. The consequences of reduced cooperativity on DNA binding in vivo were addressed by expressing a panel of cooperativity mutants ectopically in p53-null Saos-2 cells [52,75]. Comprehensive genome-wide ChIP-seq analysis uncovered several important aspects. First, the number of p53-bound sites within the genome decreased in parallel with the reduction in cooperativity: whereas approximately 5000 binding peaks were detected for wild-type p53, only 1667 sites were occupied by the E180R mutant and just 88 by R181E. Intriguingly, co-expression of the two “low cooperativity” mutants E180R and R181E, which showed in vitro a DNA binding capacity exceeding that of the wild-type protein, resulted in even stronger association with DNA and a larger number of binding peaks than wild-type p53. Second, analysis of peak sequences showed that the sequence-specificity of DNA binding increased with reduction in cooperativity: while DNA binding of mutants with reduced cooperativity was largely limited to sites matching the consensus sequence, wild-type and high cooperativity p53 (E180R+R181E) bound DNA in a more promiscuous manner, resulting in binding to non-canonical REs, in which half-sites were often separated by larger spacers and/or contained CAAG and CTAG in the middle. [53]. The fact that binding to non-canonical REs is more strongly dependent on cooperativity than binding to consensus sites is important for understanding the consequences of cooperativity mutations on p53′s function as a tumor suppressor.

### 4.2. Cooperativity Mutations Affect Transcriptional Activity of p53

**Figure 4:**
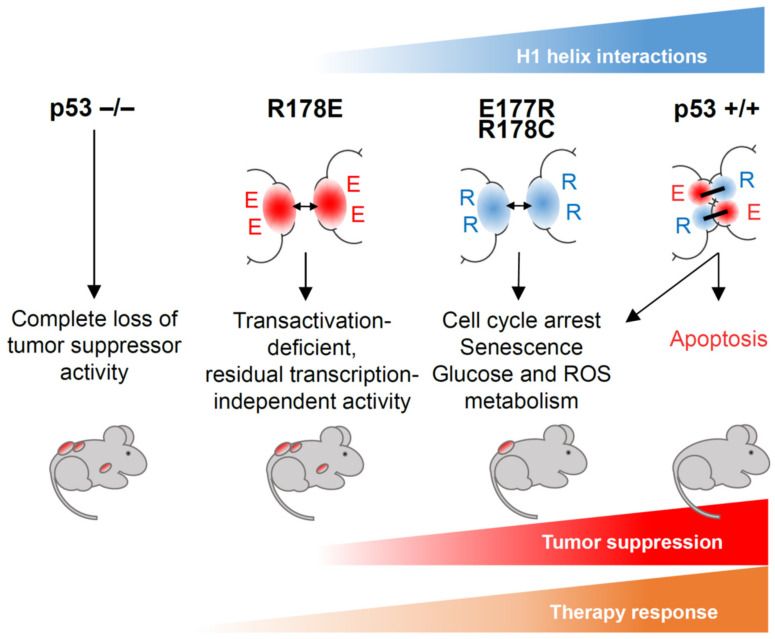
Cooperativity mutant mouse models show that the strength of interaction between H1 helices determines the functional activity of p53. Loss of cooperativity in p53R178E (p53EE) mouse leads to a phenotype similar to p53 knock-out, but residual transcription-independent activity provides a better therapy response in comparison to p53-null. Reduced cooperativity (E177R, R178C) results in compromised apoptosis but retained pro-survival functions of p53.

Stable interaction of p53 with REs is a prerequisite for its transactivation function, but differences in RE structure determine binding affinity and influence efficiency and dynamics of target gene activation [45,76,77]. In vitro experiments showed that consensus-like binding sites that are present in promoters of cell cycle regulatory genes (e.g., SFN/14-3-3σ, *CCNG1*/Cyclin G1, *GADD45A*, *CDKN2A*/p21) are bound by wild-type p53 with high affinity, whereas the binding affinity to less conserved REs that are found in regulatory elements of proapoptotic genes varies dramatically [34,76,78]. This led to the hypothesis that binding affinity can be a part of the decision-making mechanism that directs the p53-dependent transcriptional program toward survival or cell death [76,78]. ChIP experiments with p53 cooperativity mutants provided evidence supporting this hypothesis. As mentioned above, p53 variants with reduced cooperativity preferentially bound high affinity REs, whereas strong cooperativity made p53 more promiscuous and allowed efficient binding to low affinity sites [53]. Functional annotation of genes located in the vicinity of p53 binding sites occupied by high or low cooperativity p53 uncovered a strong correlation: sites occupied by low cooperativity variants were enriched for cell cycle and survival genes, whereas the non-canonical sites that were only bound by high cooperativity p53 were associated with apoptosis genes [52,53,79]. This indicated that cooperativity-reducing mutations not only narrow the spectrum of regulated genes but also favor a transcriptional pro-survival program. Gene expression analysis showed a different degree of transcriptional defect in cooperativity mutants: E180R renders p53 unable to induce pro-apoptotic targets but spares its proficiency in regulation of genes involved in cell cycle, senescence, and metabolism; R181E has a more severe impact because it completely disables p53′s transcriptional activity [52,53,74,80]. Hence, these mutations can separate p53 functions: E180R segregates non-apoptotic transcriptional programs governed by p53, whereas R181E isolates transcription-independent activities. Importantly, as similarly observed in vitro, the combination of the two different mutant alleles in one cell leads to full restoration of transactivation and rescue of apoptosis [53,80]. Other H1 helix mutants such as E180K, R181L, R181H, R181C, and R181P (which are found in human cancers) showed similarly reduced transcriptional activity toward p53 target genes, particularly ones involved in apoptosis [25,52,81]. Thus, the strength of interaction between H1 helices determines the spectrum of regulated genes and shapes the functional activity of p53, giving rise to intriguing separation-of-function effects (Figure 4).

## 5. Cooperativity and Tumor Suppression

Cooperativity mutations that affect the critical Glu180 and Arg181 salt bridge residues are detected in tumors, suggesting an importance of p53 DNA binding cooperativity for tumor suppression [16,82]. Experimental data obtained on different mouse models with altered cooperativity strongly support this notion.

Despite significant differences in primary sequence between human and mouse p53, the 3D structure of their DBDs, including their protein–protein contact interface, is very similar [46,83]. Genetically modified mouse models were therefore successfully used for studying p53 functions and consequences of its inactivation in cancer [84]. Several mouse models were established to investigate the physiological role of p53 DNA binding cooperativity (Figure 4). Initially, knock-in mice with cooperativity mutations E177R and R178E (corresponding to human E180 and R181, respectively) targeted into the endogenous *Trp53* locus were described [74,80,85]. Although these mutations have not yet been identified in cancer, they have two important features: first, the precise mechanism of action—reduction of cooperativity leading to weakened DNA binding—is well characterized for these mutations; second, as suggested by structural data and confirmed in multiple experiments, these mutations do not affect p53 monomer structure. These features make E177R and R178E knock-in mice very valuable tools for studying different aspects of p53 functions in vivo. Recently the cancer-associated cooperativity mutation R181C has been modeled using the R178C knock-in mouse [81]. Together, these studies elucidated the significance of cooperativity for p53-mediated tumor suppression.

### 5.1. Trp53^E177R^ Mouse

The E177R mutation makes mouse p53 deficient in regulation of proapoptotic genes (such as Puma, Bax, Noxa), similar to the human E180R mutant. Embryonic fibroblasts (MEFs), splenocytes, and thymocytes isolated from homozygous p53^E177R^ knock-in mice showed no apoptosis in response to DNA damage. Concomitantly, no apoptosis was observed in radiosensitive tissues (spleen, thymus, intestine, developing brain) after ionizing irradiation (IR) in vivo. On the other hand, E177R retained proficiency in transcriptional activation of target genes responsible for regulation of cell proliferation and senescence, being able to trigger cell cycle arrest in tissues after IR, block proliferation in response to genotoxic treatment, and mount oncogene- or stress-induced senescence in MEFs [80]. Reduced cooperativity did not abolish p53-dependent regulation of genes involved in antioxidant defense, autophagy, and metabolism, which protected cells from oxidative DNA damage and suppressed early spontaneous development of thymic lymphoma. Yet, the tumor suppressive power of p53 was strongly compromised, as demonstrated by the inability to counteract progression of Eμ-Myc-induced B-lymphoma and the high incidence of other spontaneous tumors, which resulted in reduced survival of knock-in mice [80]. These findings indicate that p53-mediated tumor suppression relies on multiple context-dependent mechanisms and emphasize the importance of DNA binding cooperativity for full p53-mediated tumor suppression. Importantly, the reduced spectrum of regulated genes and the resulting tumor susceptibility phenotype of E177R mice are very reminiscent of many partial LOF mutations detected in human tumors, which suggests this mouse model as a useful prototype for studying cancers with partially disabled p53.

Interestingly, the *Trp53*^R172P^ knock-in mouse, which models the human R175P mutation, has a hypomorphic phenotype very similar to the *Trp53*^E177R^ mouse. Despite being apoptosis-deficient and cancer-prone, the R175P mutant can induce cell cycle arrest, maintain chromosomal stability, and counteract the development of certain tumor types such as Kras-driven pancreatic adenocarcinoma [86,87]. The mechanism underlying the hypomorphic nature of the R175P mutant is yet unclear. R175 is located immediately adjacent to the zinc-chelating residue R176 and, as such, R175 mutations weaken metal-binding affinity to a variable extent, depending on the exact type of mutation [67,72,88]. Zinc binding is crucial for correct positioning of the DNA binding surface formed by the L2 and L3 loop. A simple explanation for the hypomorphic nature of the R175P mutation is therefore a subtle and less severe disruption of the p53–DNA interface than observed for the more frequent R175H. However, embedded in the L2 loop is the H1 helix, which makes it tempting to speculate that small perturbations in the Zn-coordination sphere caused by R175P affect the H1 helix salt bridges and thereby give rise to a similar phenotype as a direct salt bridge mutation. Further structural studies on the consequences of various p53 mutations on the ternary structure of p53 are required to resolve this.

### 5.2. Trp53^R178C^ Mouse

The p53^R178C^ mouse was generated as a model for the human cancer-related *TP53* R181C mutation, which has been detected in the hereditary Li-Fraumeni cancer predisposition syndrome (LFS) and various spontaneous cancer entities [81]. R181C was one of the first *TP53* germline mutations described and one of the most frequently found in LFS patients with breast cancer at or before the age of 40 years [89]. Like other cooperativity mutations, R178C affects DNA binding and transcriptional functions of p53. The genome-wide analysis of R178C DNA binding demonstrated that the number of genomic sites occupied by the protein is strongly reduced with a binding preference for REs that are bound with low cooperativity, very similar to the E177R mutant. For instance, no binding to the promoter of pro-apoptotic Bax was detected, whereas recruitment of R178C to p21 and Mdm2 promoters was only decreased. This was associated with reduced expression of p53 target genes and compromised apoptosis and with retained functions in regulation of senescence and metabolism, again remarkably reminiscent of E177R. Interestingly, R178C mutant mice showed a decreased white adipose tissue mass, higher lipolytic activity, and increased expression of lipid metabolism genes, particularly *Adrb3*, identified as a direct p53 target. Importantly, the homozygous knock-in mice displayed a shortened lifespan and were tumor prone, further confirming the essential role of DNA binding cooperativity for tumor suppression. [81].

### 5.3. Trp53^R178E^ Mouse

The human R181E mutation resulted in virtually complete loss of DNA binding and loss of direct transcriptional regulation of p53 target genes [53]. In agreement with these data, the homologous R178E mutation in mice led to complete deficiency in chromatin binding and transactivation [74]. As a result, functions of p53 in driving transcription-mediated apoptosis and cell cycle arrest in response to genotoxic stress were lost, although ROS-induced senescence seemed to be at least partially operational, presumably via transcription-independent mechanisms. Homozygous R178E mice succumbed to spontaneous cancer with similar disease latency and penetrance as p53 knock-out mice, which was also true for Eμ-Myc-induced lymphoma and acute myeloid leukemia (AML) driven by AML1/ETO9a and Nras^G12D^. Failure to delay the development of spontaneous and oncogene-induced cancer suggested a complete absence of tumor-suppressive activity upon loss of DNA binding cooperativity. However, different from the p53 knock-out, the R178E mutant did not rescue the embryonic lethality caused by genetic inactivation of the p53 negative regulator Mdm2. Moreover, in MEFs and upon overexpression in human lung cancer cells, R178E elicited pronounced apoptosis under combined treatment with doxorubicin and the Mdm2 inhibitor Nutlin-3a. This unexpected lethal activity, which was not associated with transcriptional activation of proapoptotic genes, was explained by a cytoplasmic fraction of the R178E mutant that localized to mitochondria, associated with Bcl-2 family proteins and triggered mitochondrial outer membrane permeabilization (MOMP) [74]. Mechanisms of non-transcriptional apoptosis driven by p53 are well described, but due to the lack of suitable in vivo models its role in tumor suppression and therapy responses had remained elusive [90]. The R178E cooperativity mutant mouse has provided first insight into transcription-independent p53 functions during cancer therapy. In two therapeutic models for B-cell lymphoma and AML, the R178E mutant provided a superior response to chemotherapy and significantly extended mouse survival as compared with a p53-null setting [91]. This finding indicates that, although non-transcriptional functions of R178E are insufficient to suppress tumor progression, they can be successfully engaged by therapy and improve clinical responses. Whether this is true for other p53 cooperativity and LOF mutations found in human cancer needs further investigation.

## 6. Mechanisms of Regulation of DNA Binding Cooperativity

The possibility to separate pro-survival and pro-apoptotic p53 activity via modulation of cooperativity raised the important question of whether and how cooperativity is regulated. p53 undergoes multiple post-translational modifications (PTM) that influence its interaction with DNA and other proteins and determine stability, cellular localization, and transcriptional and transcription-independent activities [92,93]. The majority of modifications with known regulatory functions map to the N- and C-terminal domains of p53. In addition, proteomic studies detected several modified sites also in the DBD, many of which still remain poorly characterized [94,95].

### 6.1. H1 Helix Phosphorylation

**Figure 5:**
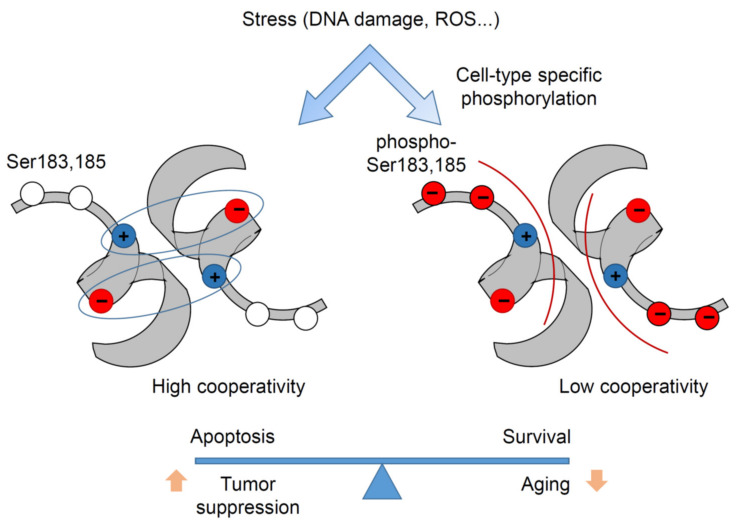
Regulation of DNA binding cooperativity via serine phosphorylation. Left—In the absence of Ser183, Ser185 phosphorylation double salt bridges between Glu180 (red) and Arg181 (blue) of antiparallel H1 helices (schematically shown in gray) determine strong cooperativity, which allows p53 to execute a full-blown response to stress (e.g., DNA damage and ROS) and to effectively suppress tumorigenesis. Right—Upon phosphorylation of Ser183, Ser185 ionic bonds are weakened, leading to reduced cooperativity. This shifts p53 activity toward pro-survival programs (cell cycle arrest, senescence), prevents excessive cell death of stem cells, and protects against premature aging.

In the human p53 DBD, there are two serine residues, S183 and Ser185, adjacent to the Glu180-Arg181 double salt bridge, which were detected to be phosphorylated in several mass-spectrometry studies [94,95,96,97]. Serine is the most common substrate of many dual-specificity kinases involved in signal transduction, and serine phosphorylation is a well-known mechanism for regulating conformational changes and protein–protein interactions [98]. Phosphorylation sets a negative charge to the uncharged serine, which can affect electrostatic interactions in the vicinity. For example, it has been shown that serine phosphorylation leads to dissociation of a salt bridge and triggers change of protein partners by Raf kinase inhibitory protein (RKIP) [99]. In the context of the p53 intra-dimer interaction interface created by the H1 helices, phosphorylation of Ser183 and Ser185 alleviates the electrostatic attraction between monomers and thus weakens cooperativity. Notably, p53 Ser183 is conserved in almost all mammals, whereas Ser185 is absent in some species such as rodents [55,100]. Mutations at these codons are extremely rare in cancer (about 0.04% for S183 and 0.09% for S185), which is 3- to 8-fold lower than expected by chance, suggesting that phosphorylation at these sites might be required for tumorigenesis. Some studies suggested that Aurora B inhibits p53 via S183 phosphorylation [96,97]. Phospho-mimetic (S183D, S185D) and phospho-deficient (S183A, S185A) mutants were used to investigate the impact of serine phosphorylation on DNA binding cooperativity, revealing that phospho-mimetic mutations reduce p53 DNA binding and transactivation. Colorectal HCT116 cancer cells with CRISPR-engineered phospho-mimetic S183D and S185D mutations at the endogenous *TP53* locus demonstrated reduced expression of p53-regulated genes, increased resistance to doxorubicin, and a marked drop in apoptosis, closely resembling the cooperativity mutant E180R phenotype. In contrast, isogenic S183A and S185A double-mutant cells displayed enhanced transcription of p53 targets and increased apoptosis after doxorubicin treatment [100]. Furthermore, phosphorylation-deficient *Trp53*^S180A^ knock-in mice with a serine-to-alanine substitution at Ser180 (homolog to human S183) have been generated. MEFs isolated from these mice showed moderately increased DNA binding of p53 and enhanced expression of p53 target genes. While the effects at the organismal level were highly tissue- and cell-type dependent, the hematopoietic compartment of knock-in mice was exquisitely sensitive and failed to properly regenerate after DNA damage. S180A mice displayed increased resistance to spontaneous and oncogene-induced tumorigenesis but suffered from a shortened lifespan due to a general loss of fitness and increased risk of age-related diseases of the respiratory and cardiovascular system. Together these data provide evidence that serine phosphorylation can reversibly regulate p53 DNA binding cooperativity and determine transcriptional selectivity in a cell type-dependent manner (Figure 5).

### 6.2. Contribution of CTD to Cooperativity

The CTD is intrinsically disordered but can adopt different local structures upon interaction with other proteins [101,102]. Such structural flexibility is characteristic of regulatory domains involved in signal transduction and allows fast, low-affinity, and therefore reversible but highly specific protein interactions [103]. Being a substrate for numerous protein-modifying enzymes, the CTD is subject to multiple post-translational modifications, such as acetylation, phosphorylation, methylation, neddylation, sumoylation, and ubiquitination, which determine stability, cellular localization, interaction partners, and functional activity of p53 [6,104,105]. p53 mutants that lack the CTD show impaired DNA binding and compromised transcriptional activity [106,107,108]. The CTD can bind DNA in a sequence-independent manner [109] and can promote linear diffusion of p53 on double-stranded DNA fragments in vitro [106,110,111]. Acetylation of conserved C-terminal lysine residues K370, K372, K373, K381, K382, and K386 by CBP/p300 influences the interaction of the CTD with DNA [112,113] and proteins [114,115,116] and the recruitment of transcriptional co-factors to p53 REs [117,118]. Although initial reports about the role of CTD for sequence-specific binding were controversial, it has been shown that upon interaction with long DNA fragments, the CTD can enhance sequence-specific DNA binding [119,120]. This effect is particularly evident in the context of imperfect, non-canonical REs and can be fine-tuned by CTD acetylation [59,121]. Mechanistically, the CTD was found to induce conformational changes in the DBD itself that protect the H1 helix-containing region from proteolytic cleavage, consistent with a model where the CTD facilitates H1 helix interactions within the tetramer to strengthen DNA binding cooperativity and p53-DNA complex stability at non-canonical REs [59]. Moreover, the negative impact of CTD deletion on DNA binding was largely reversed by the cooperativity-enhancing E180R;R181E double-mutation, providing further support for this intriguing intramolecular crosstalk between CTD and H1 helix in fine-tuning DNA binding cooperativity and target gene selectivity [59].

## 7. Cooperativity Mutations and Therapy

The high prevalence of p53 mutations in 50% of cancers obviously makes mutant p53 an attractive therapeutic target. However, the broad spectrum of missense mutations that affect p53 and the variability of functional consequences of these mutations impede development of a common targeting strategy. For hotspot mutants, which have completely lost their tumor suppressive functions and exert clear dominant-negative and pro-tumorigenic GOF activities, targeting therapies have been developed that aim to restore wild-type function or at least abolish the oncogenic properties of the mutant protein [64,122].

In the case of cooperativity mutants, these strategies need to be refined. First, tumorigenesis in *Trp53*^E177R/+^ and *Trp53*^R178E/+^ mice is significantly delayed compared with *Trp53*^E177R/−^ and *Trp53*^R178E/−^ mice, respectively, arguing against a dominant-negative activity of cooperativity mutants during tumor development [74,80]. Second, in contrast to mice with p53 hotspot mutations, there is no evidence for increased metastasis associated with cooperativity mutants, suggesting an absence of pro-metastatic GOF properties [74,80]. Third, cooperativity mutants retain some transcriptional and/or non-transcriptional functions of the wild-type protein, and these residual activities have been shown to enhance chemotherapy responses, leading to prolonged survival of treated mice [74,80,81]. In the absence of strong evidence for oncogenic properties of cooperativity mutants, mutant p53 degrading or blocking therapies, developed for p53 hotspot mutants, are likely inefficient. Moreover, as such strategies would also abolish the residual tumor suppressive functions of the mutant, they might even be counterproductive. Instead, cooperativity mutant cancers might be treated more successfully by exploiting and boosting the residual tumor suppressive activities of the mutant protein.

### 7.1. Boosting Mitochondrial Apoptosis Using BH3-Mimetics

Cooperativity mutants were found to retain transcription-independent apoptotic functions [52,74,85]. Similar to other p53 mutants, cooperativity mutant p53 accumulates in tumors, and a substantial part of it seems to reside on mitochondria even in non-stressed conditions and to sensitize cells to MOMP by BH3-only proteins such as BID [74,85]. Upon DNA damage, mitochondrial localization further increased, and interaction with Bcl-2, Bcl-xL, and Bak was observed [74]. Mdm2 inhibitors increased protein levels of cooperativity mutants even further and synergized with cytotoxic drugs to induce strong apoptosis in cells resistant to either single treatment. Whereas combination of Mdm2 inhibitors with doxorubicin or other cytotoxic drugs is therapeutically limited because of severe side effects [123,124], the use of BH3 mimetics for lowering mitochondrial apoptotic threshold may be an alternative. These drugs such as ABT-737, ABT-199 (venetoclax), or ABT-263 (navitoclax) directly promote MOMP by sequestering antiapoptotic proteins Bcl-2 and Bcl-xL. Numerous preclinical tumor models and clinical studies confirmed the efficiency of BH3 mimetics as single agents or in combination with standard anti-cancer drugs [125]. Importantly, it has been shown that constitutive expression of wild-type p53 can sensitize cells to ABT-737 via transcription-independent mechanisms [126], strongly suggesting that mitochondrial apoptotic priming by cooperativity mutants generates a druggable vulnerability to BH3 mimetics.

### 7.2. ROS-Mediated Therapy

The most advanced mutant p53 targeting compound APR-246 (PRIMA-1^MET^; eprenetapopt), which demonstrates promising results in pre-clinical and clinical investigations [127,128], is believed to switch mutant p53 into a wild-type conformation via covalent binding to multiple cysteines within p53′s DBD [129,130]. APR-246 activates transcription of p53 target genes, induces apoptosis, and suppresses growth of human cancer xenografts in a mutant p53-dependent manner [131,132,133] and synergizes with other chemotherapeutic drugs [134,135,136]. Different from structural mutants, DNA contact and cooperativity mutants have a wild-type conformation and would not be expected to profit from APR-246. However, since DNA contact mutants such as R273H are sensitive to APR-246, other mechanisms likely contribute to the therapeutic efficacy of APR-246. For example, APR-246 was found to trigger oxidative stress and decrease glutathione levels leading to lipid peroxidation and ferroptosis [137,138,139,140]. Notably, mutant p53 potentiated susceptibility of cancer cells to oxidative stress and ferroptosis by inhibiting the Xc-system, binding to the NRF2 transcription factor and reducing glutathione synthesis, providing an additional explanation for the APR-246 vulnerability of p53 mutant cells [141]. Interestingly, the cooperativity mutant R180E also reduced expression of NRF2-regulated genes and increased cellular ROS levels, suggesting that APR-246 might be used to elicit therapeutic responses also in cooperativity mutant tumors [74].

### 7.3. Targeting Senescence

Cellular senescence, considered as an initial barrier to malignant transformation, can promote tumor progression at later stages of tumorigenesis and support therapy resistance [142]. In breast cancer, senescence induced by wild-type p53 upon chemotherapy compromises treatment efficiency and results in inferior survival [143,144,145]. Therapy-induced senescence not only protects cancer cells from apoptosis under treatment but also contributes to the development of stemness and promotes disease relapse [143]. Most cooperativity mutants are impaired in transcriptional apoptosis but often retain the ability to induce senescence, which might interfere with cytotoxic therapies such as radio- or chemotherapy. Molecules that specifically target senescent cells, so-called senolytics, may therefore be an option to improve the therapy efficiency for cancers with cooperativity mutations. A recent study demonstrated that the combination of ABT-263 with doxorubicin efficiently overcomes resistance to apoptosis in breast cancer cells, driven to senescence by wild-type p53 [144]. In addition to ABT-737 and ABT-263, other compounds with different mechanisms of action are reported to possess senolytic activity—for example, the kinase inhibitor dasatinib, the flavoinoid quercetin, the heat shock protein Hsp90 inhibitors geldanamycin and tanespimycin, or the histone deacetylase inhibitor panobinostat [145]. It would therefore be interesting to investigate whether cancers with cooperativity mutant p53 (and maybe other non-hotspot LOF mutants with preserved pro-senescence activity) are sensitized to standard cytotoxic chemotherapy by senolytic drugs.

## 8. Conclusions

DNA binding cooperativity mutations that strike the H1 helix are distinct from structural or contact mutations affecting the p53 DNA-binding domain. They are bona fide cancer mutations associated with both sporadic and hereditary tumor types. Unlike the majority of other non-hotspot p53 mutations, cooperativity mutations have a known mode of action, yielding unique partial-LOF or separation-of-function phenotypes that mechanistically distinguish transcription-dependent from independent and pro-apoptotic from pro-survival functions. This makes cooperativity mutations exceptionally valuable for understanding p53 tumor suppressor functions and for developing treatment approaches for cancers with p53 partial-LOF mutations.
